# Supplementary material for: Mendelian randomization with proxy exposures: challenges and opportunities
Source: Genetics. 2025 Sep 26;231(4):iyaf210. doi: 10.1093/genetics/iyaf210 (PMC12693524; doi:10.1093/genetics/iyaf210)
Supplement: iyaf210_Supplementary_Data [file iyaf210_supplementary_data.zip › Supplementary_Figs._1-9_GENETICS-2025-308594.pdf]

# Supplementary figures

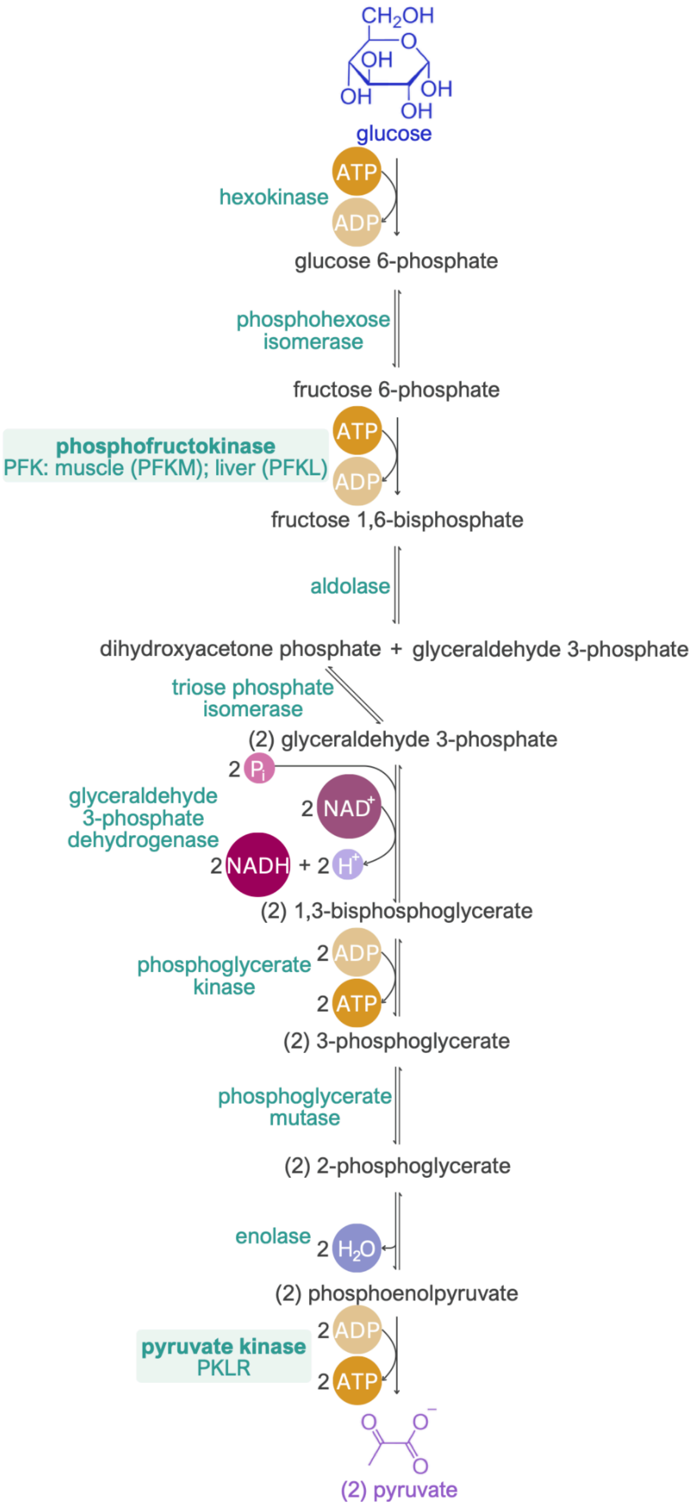


**Supplementary Fig. 1**. Diagram of the glycolysis pathway.

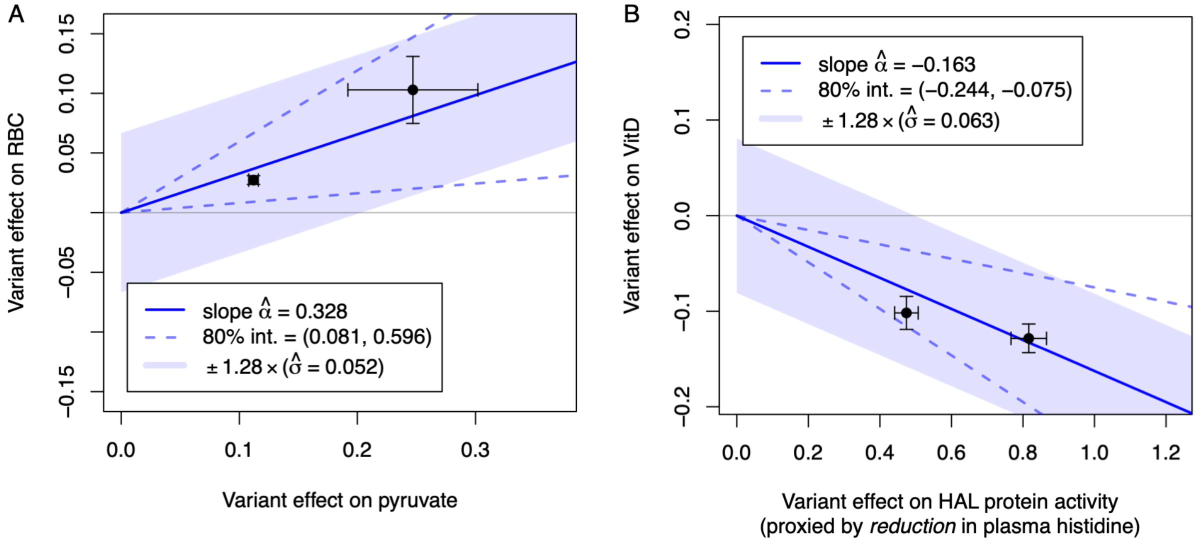


**Supplementary Fig. 2.** Replication of the *cis*-MR analysis using metabolite data from the Estonian Biobank. (A) Replication of the *cis*-MR analysis between proxied glycolysis pathway activity and RBC count. Variant effect on pyruvate has now been measured in the Estonian Biobank (n = 185,352) while variant effect on RBC count has still been measured in the UK Biobank as in the primary analysis (Fig. 2d). The two variants included in the analysis are the *PKLR* missense variant 1_155291918_G_A and the *PFKM* missense variant 12_48118502_C_A. The other two variants were not imputed in the Estonian Biobank due to their low allele frequency. (B) Replication of the *cis*-MR analysis between proxied HAL activity and plasma vitamin D levels. The variant effect on histidine (exposure) has now been measured in the Estonian Biobank (n = 185,352) while the variant effect on vitamin D has still been measured in the UK Biobank as in the primary analysis. The two *HAL* missense variants included in the analysis are 12_95977953_C_T (rs61937878) and 12_95986106_C_T (rs117991621). LD between the variants is shown in Supplementary Table 2.

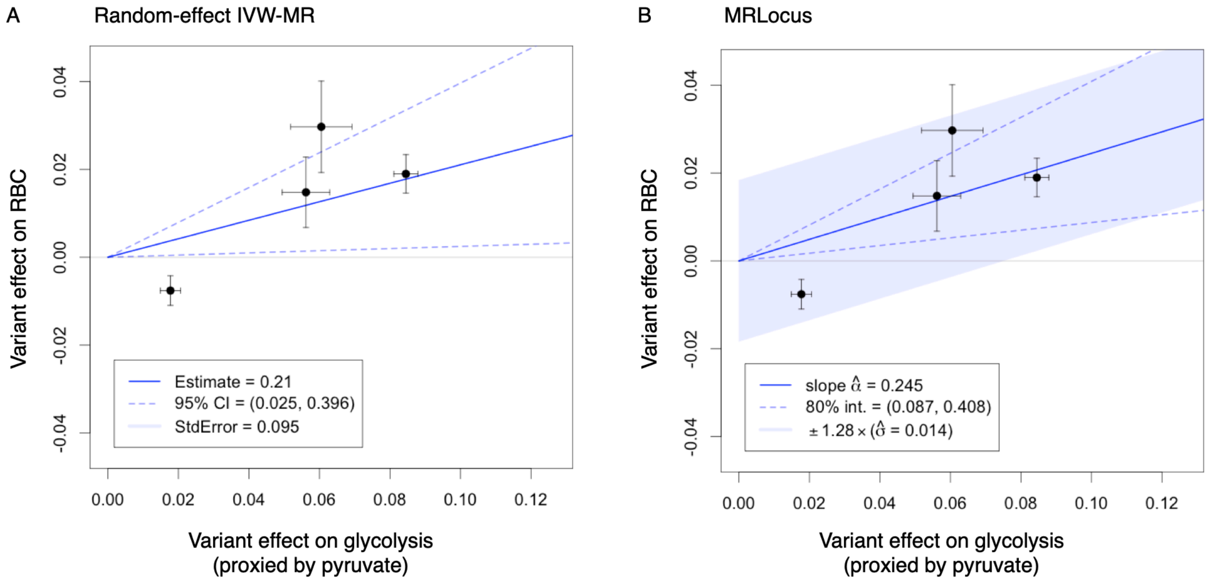


**Supplementary Fig. 3.** *cis*-MR at the *PKLR* locus using greedily pruned variants. Here, four instruments (rs75925257, rs115736167, rs72704114 and rs141119689) were selected among variants with MAF > 0.01 using a greedy LD pruning approach (r2 < 0.01, see Methods). The two *PKLR* missense variants were not included due to their low allele frequency. (A) pyruvate vs RBC count, MR-IVW (B) pyruvate vs RBC count, MRLocus. RBC - red blood cell.

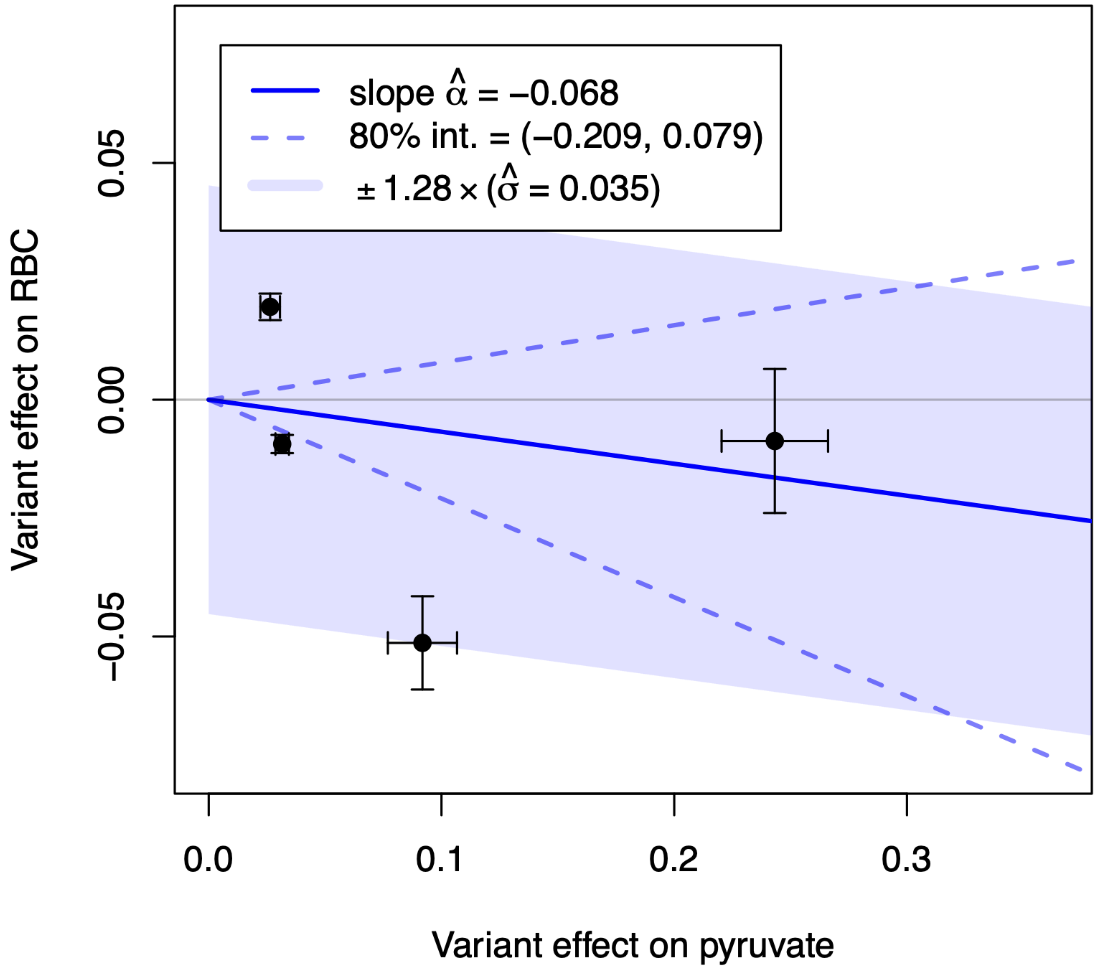


**Supplementary Fig. 4.** Mendelian randomisation between plasma pyruvate (exposure) and red blood cell count (RBC) (outcome) using missense variants outside of the glycolysis pathway (*GCKR*, *NDOR1*, *AMPD3*, *PDK3*) as instruments.

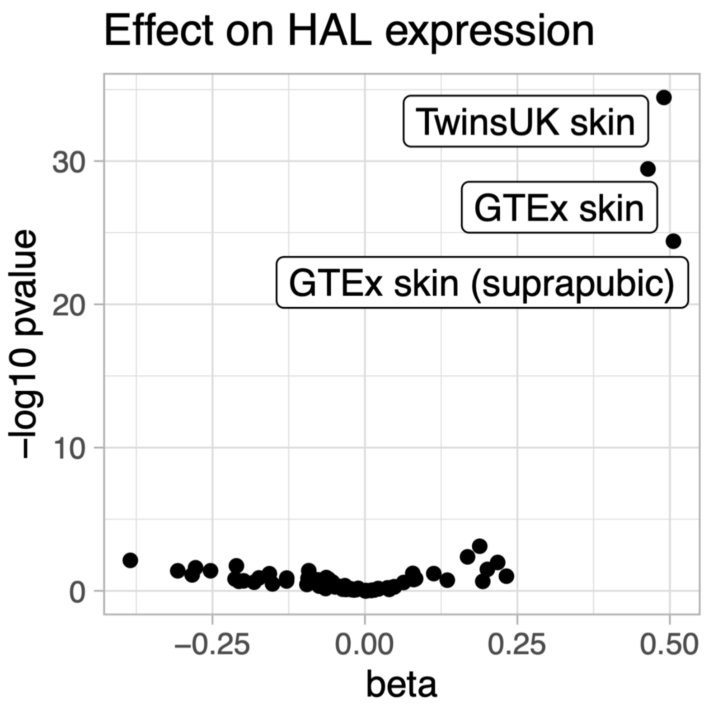


**Supplementary Fig. 5**. Volcano plot of the vitamin D lead variant effect on *HAL* expression across 127 eQTL Catalogue release 6 datasets.

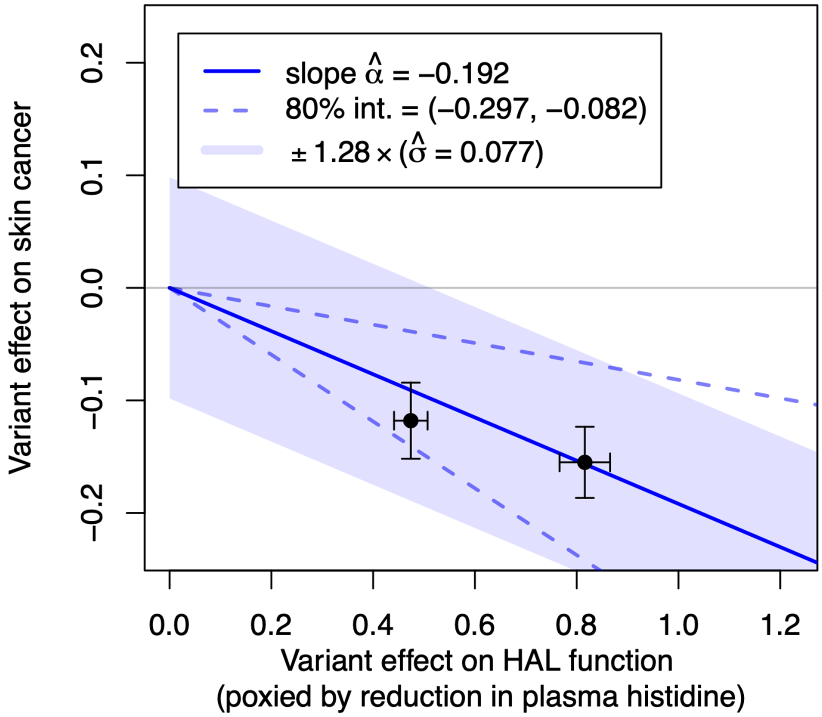


**Supplementary Fig. 6.** MR analysis examining the relationship between proxied HAL protein activity and skin cancer risk. Variant effect on plasma histidine was measured in the UK Biobank and variant effect on skin cancer was extracted from the GWAS meta-analysis of UK Biobank, FinnGen and Million Veterans Program (MVP) cohorts (https://mvp-ukbb.finngen.fi/). The two *HAL* missense variants included in the analysis are 12_95977953_C_T (rs61937878) and 12_95986106_C_T (rs117991621). LD between the variants is shown in Supplementary Table 2. The individual betas and standard errors are shown in Supplementary Table 3.

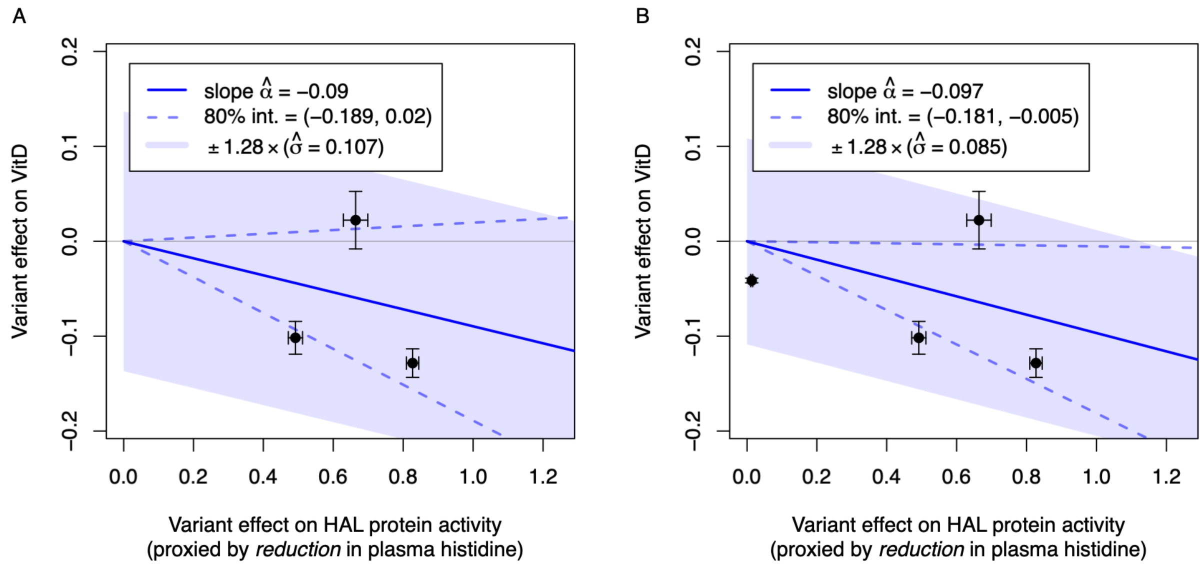


**Supplementary Fig. 7.** MR analysis between HAL function and vitamin D, using all three fine-mapped missense variants as instruments. (A) MR analysis with the three *HAL* missense variants [12_95977953_C_T (rs61937878), 12_95986106_C_T (rs117991621) and 12_95994812_G_A (rs143854097)] included in the analysis. (B) MR analysis with the HAL eQTL variant added to the three missense variants. Pairwise LD between the variants is shown in Supplementary Table 2.

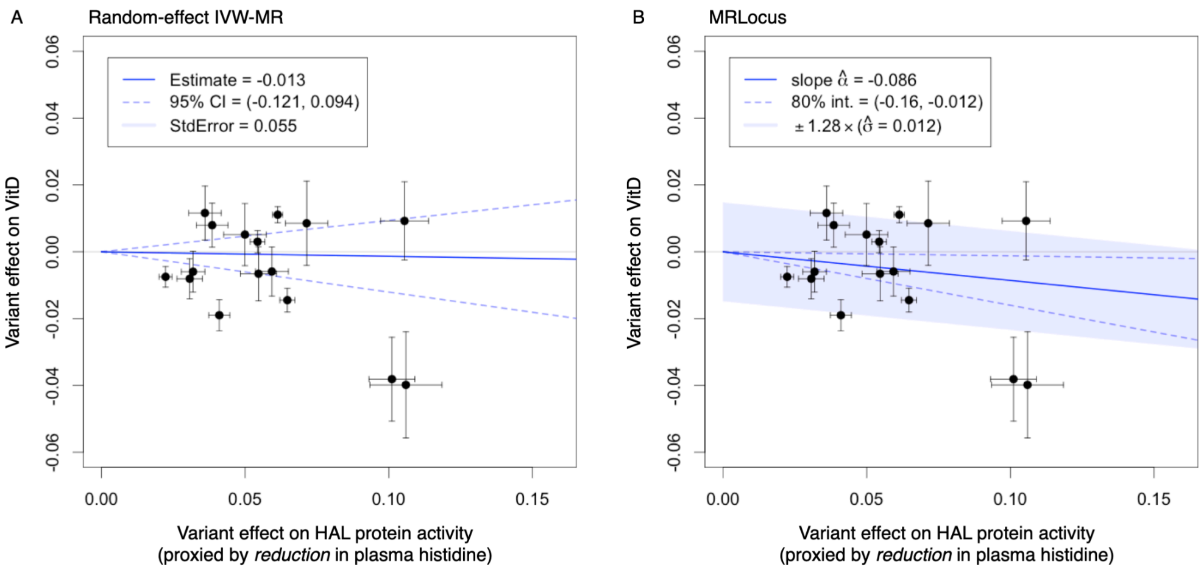


**Supplementary Fig. 8.** *cis*-MR at the *HAL* locus with greedy LD pruning to select instruments. Instruments were selected among variants with MAF > 0.01 using a greedy LD pruning approach ($r^2 < 0.01$). The three *HAL* missense variants were not included due to their low allele frequency. (A) MR analysis using the multiplicative random-effect inverse-variance weighted MR (IVW-MR) method. (B) MR analysis using the MRLocus method.

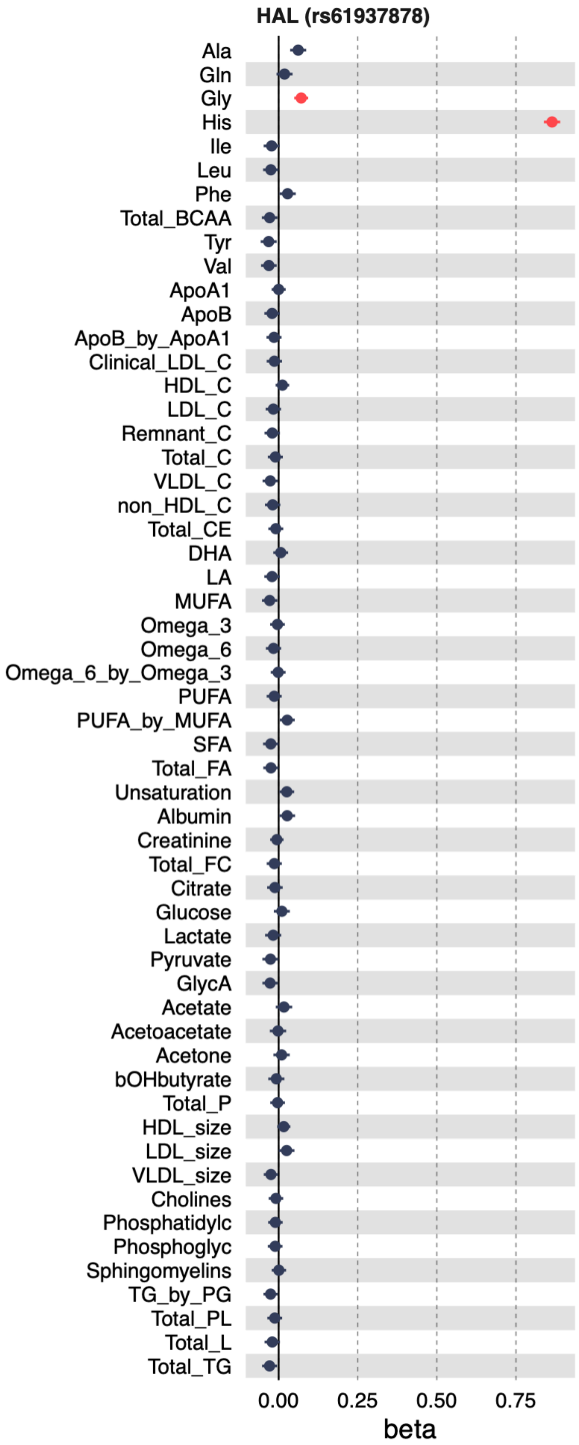


**Supplementary Fig. 9.** Association effect sizes and 95% confidence intervals for the *HAL* missense variant rs61937878 across 56 metabolic traits in the UK Biobank + Estonian Biobank meta-analysis (Tambets *et al.* 2025)**.** The two genome-wide significant associations ($p < 5x10^{-8}$) are with histidine (His) and glycine (Gly). The absolute variant effect on glycine (beta = 0.071; $p = 2.5x10^{-10}$) is even smaller than the variant effect on vitamin D (beta = -0.12841), likely reflecting an indirect pleiotropic effect.
